# Pectic polysaccharides are attacked by hydroxyl radicals in ripening fruit: evidence from a fluorescent fingerprinting method

**DOI:** 10.1093/aob/mcv192

**Published:** 2016-02-09

**Authors:** Othman B. Airianah, Robert A. M. Vreeburg, Stephen C. Fry

**Affiliations:** The Edinburgh Cell Wall Group, Institute of Molecular Plant Sciences, The University of Edinburgh, Daniel Rutherford Building, The King’s Buildings, Max Born Crescent, Edinburgh EH9 3BF, UK

**Keywords:** Fruit, ripening, cell wall, pectic polysaccharides, hydroxyl radicals, non-enzymic scission, fluorescent labelling, fingerprint compounds

## Abstract

**Background and aims** Many fruits soften during ripening, which is important commercially and in rendering the fruit attractive to seed-dispersing animals. Cell-wall polysaccharide hydrolases may contribute to softening, but sometimes appear to be absent. An alternative hypothesis is that hydroxyl radicals (^•^OH) non-enzymically cleave wall polysaccharides. We evaluated this hypothesis by using a new fluorescent labelling procedure to ‘fingerprint’ ^•^OH-attacked polysaccharides.

**Methods** We tagged fruit polysaccharides with 2-(isopropylamino)-acridone (pAMAC) groups to detect (*a*) any mid-chain glycosulose residues formed *in vivo* during ^•^OH action and (*b*) the conventional reducing termini. The pAMAC-labelled pectins were digested with Driselase, and the products resolved by high-voltage electrophoresis and high-pressure liquid chromatography.

**Key Results** Strawberry, pear, mango, banana, apple, avocado, *Arbutus unedo*, plum and nectarine pectins all yielded several pAMAC-labelled products. GalA–pAMAC (monomeric galacturonate, labelled with pAMAC at carbon-1) was produced in all species, usually increasing during fruit softening. The six true fruits also gave pAMAC·UA-GalA disaccharides (where pAMAC·UA is an unspecified uronate, labelled at a position other than carbon-1), with yields increasing during softening. Among false fruits, apple and strawberry gave little pAMAC·UA-GalA; pear produced it transiently.

**Conclusions** GalA–pAMAC arises from pectic reducing termini, formed by any of three proposed chain-cleaving agents (^•^OH, endopolygalacturonase and pectate lyase), any of which could cause its ripening-related increase. In contrast, pAMAC·UA-GalA conjugates are diagnostic of mid-chain oxidation of pectins by ^•^OH. The evidence shows that ^•^OH radicals do indeed attack fruit cell wall polysaccharides non-enzymically during softening *in vivo*. This applies much more prominently to drupes and berries (true fruits) than to false fruits (swollen receptacles). ^•^OH radical attack on polysaccharides is thus predominantly a feature of ovary-wall tissue.

## INTRODUCTION

### Ripening: hydrolytic vs. oxidative

Fruit ripening is often accompanied by changes in flavour, odour, colour and texture which are attractive to the animals that will disperse the seeds. In particular, many berries, drupes and pomes soften during ripening owing to changes in cell wall organization. Plant cell walls are complex networks based on cellulose microfibrils, partly tethered by hemicelluloses, with pectic polysaccharides infiltrating the rest of the wall matrix (Albersheim *et al.*, 2010; Fry, 2011*a*). During fruit softening, the matrix polysaccharides, especially the pectins, often become more readily extractable and/or decrease in molecular weight, indicating depolymerization, e.g. in avocado, plum, mango, banana and tomato (Huber and O’Donoghue, 1993; Prasanna *et al.*, 2003; Ali *et al.*, 2004; Ponce *et al.*, 2010; Basanta *et al.*, 2014). The importance of pectic depolymerization has led to the widely held view that ripening can be regarded as principally a ‘hydrolytic’ process.

Earlier, however, Blackman and Parija (1928) had suggested that ripening involves a loss of ‘organisational resistance’, i.e. fruit cells lose the ability to maintain separate compartments owing to cellular (membrane) degeneration. Although this concept lost popularity, some workers continued to interpret ripening as a form of senescence attributable to oxidation reactions (Brennan and Frenkel, 1977) and more recently to emphasize the (possibly related) decrease in water content that occurs near the onset of ripening (Frenkel and Hartman, 2012). There are indeed similarities between physiological changes (e.g. chlorophyll loss and membrane permeabilization) occurring in a ripening fruit and in a leaf or petal approaching abscission. Lipoxygenases, which often increase during ripening (Ealing, 1994; de Gregorio *et al.*, 2000), generate hydroperoxide groups (>CH–OOH) in unsaturated fatty acid residues, accompanied by the formation of reactive oxygen species (ROS). Such lipid oxidation may permeabilize membranes, resulting in the release of certain metabolites, e.g. ascorbate (Dumville and Fry, 2003), into the apoplast (the aqueous solution that bathes the cell wall), and ROS by-products may drive other oxidative reactions.

Viewing fruit ripening as an ‘oxidative’ process is supported by evidence from several quarters. For example, in avocado (Lauraceae; Meir *et al.*, 1991) and serviceberry (Rosaceae; Rogiers *et al.*, 1998), lipid peroxidation was the earliest symptom of ripening, and tomato (Solanaceae) fruit ripening was accompanied by elevated H_2_O_2_ and the oxidation of lipids and proteins (Jimenez *et al.*, 2002). In the present study, we propose a link between oxidative agents (especially the hydroxyl radical, ^•^OH) and pectic polysaccharide degradation in softening fruit.

### Wall turnover and enzymes

Primary cell walls control the texture of fruit tissues. Although strong enough to withstand turgor pressure, walls are dynamic structures in which the polymers can be remodelled or degraded, resulting in wall loosening. Many enzymes and expansins have been described that act on cell-wall polymers, and the expression of these proteins has been correlated with fruit softening as well as cell expansion and abscission (reviewed by Franková and Fry, 2013). For example, glycanases and transglycanases cleave cell wall polysaccharides in mid-chain (Taylor *et al.*, 1993; Bewley, 1997; Lazan *et al.*, 2004; Fry *et al.*, 2008; Schröder *et al.*, 2009; Franková and Fry, 2011; Derba-Maceluch *et al.*, 2014), glycosidases release mono- or disaccharides from non-reducing termini (Fanutti *et al.*, 1991; de Veau *et al.* 1993; Hrmová *et al.*, 1998; de Alcântara *et al.*, 2006; Franková and Fry, 2012), and expansins interfere in polysaccharide–polysaccharide hydrogen bonding (Cosgrove, 2000; Harada *et al.*, 2011; Sasayama *et al.*, 2011).

Several polymer-hydrolysing enzymes have been studied in relation to fruit softening, with tomato as the most extensively studied system (Matas *et al.*, 2009; reviewed by Fry, 2017). Several wall polysaccharide-modifying enzyme activities increase, especially endo-polygalacturonase (endo-PG), cellulase, xyloglucan endotransglucosylase, β-galactosidase, pectin-methylesterase and pectate lyase. Although the link between wall-hydrolysing enzymes and fruit softening seems intuitive, tests of this as a functional relationship have often yielded contradictory evidence. A major focus has been endo-PG in tomato. This enzyme is abundant in ripe tomato fruit (Tucker and Grierson, 1982), and suppression of its expression resulted in reduced depolymerization of pectin (Smith *et al.*, 1990). Also, expression of endo-PG in the *rin* (ripening inhibitor) mutant caused an increased degradation of fruit pectins (Giovannoni *et al.*, 1989). However, both these studies failed to show a related change in tomato fruit softening; no inhibition of softening was observed in endo-PG antisense fruit, and no effect on softening was induced by the expression of endo-PG in the *rin* mutant. Moreover, other fruits, e.g. strawberry (Pose *et al.*, 2013), persimmon (Cutillas-Iturralde *et al.*, 1993) and kiwifruit (Redgwell *et al.*, 1991), show extensive pectin solubilization and/or a decrease in molecular weight even though they possess very low levels of endo-PG (e.g. Nogata *et al.*, 1993). These observations reinforce the idea that endo-PG is not necessary for fruit softening. The proposed relationship between pectin depolymerization and fruit softening was thus not strongly supported by data, although the excessive softening associated with over-ripening can be prevented by knocking out endo-PG (in the ‘Flavr Savr’ tomato; reviewed by Krieger *et al.*, 2008). Ripening is a robust phenomenon: knocking out any individual player (e.g. endo-PG) often fails to prevent normal softening.

### ^•^OH cleaves polysaccharides *in vitro*

In addition to proteins that remodel the wall, the highly reactive hydroxyl radical (^•^OH) can cause polysaccharide chain scission non-enzymically. This phenomenon is readily demonstrated in solutions of purified cell wall polysaccharides upon treatment with ascorbate in the presence of O_2_ plus traces of Cu^2+^ or Fe^3+^ (Fry, 1998; Yamazaki *et al.*, 2003; Schweikert *et al.*, 2000, 2002) and in food-related systems (Fauré *et al.*, 2012; Mäkinen *et al.*, 2012; Iurlaro *et al.*, 2014).

^•^OH can also cleave insoluble polysaccharides that are present *in situ* as structural components of the cell wall: for example, when ^•^OH was generated within the cell walls of a frozen/thawed maize coleoptile that was being held under tension, the coleoptile extended in a fashion similar to that induced by certain wall-acting proteins or in response to *in-vivo* auxin treatment (Schopfer, 2002). Likewise, *in-vitro* ^•^OH treatment of fruit cell walls of tomato (Dumville and Fry, 2003), banana (Cheng *et al.*, 2008*b*) and longan (Duan *et al.*, 2011) promoted pectin solubilization and depolymerization.

### Proposed beneficial roles of ^•^OH

These results indicate that ^•^OH, if formed in the cell wall *in vivo*, could potentially cleave polysaccharides and thereby exert physiological effects. Indeed, it has been suggested that wall loosening induced by ROS (especially ^•^OH) contributes to fruit ripening (Brennan and Frenkel, 1977; Fry, 1998; Fry *et al.*, 2001; Dumville and Fry, 2003; Cheng *et al.*, 2008*a*; Yang *et al.*, 2008; Duan *et al.*, 2011), germination (Müller *et al.*, 2009), cell expansion (Schopfer, 2001, 2002; Rodríguez *et al.*, 2002; Liszkay *et al.*, 2004) and abscission (Sakamoto *et al.*, 2008; Cohen *et al.*, 2014). It is sometimes asserted that ^•^OH, as a highly reactive ROS, must be biologically detrimental – for example causing mutations, membrane damage and protein denaturation – and that it would be advantageous for cells to prevent ^•^OH formation or to scavenge it. However, the half-life of ^•^OH within a cellular environment such as a cell wall is estimated at approx. 1 ns, allowing it to diffuse no more than approx. 1 nm (the length of two glucose residues in a cellulose chain) before reacting with some organic molecule (Griffiths and Lunec, 1996): a very short distance in the context of a primary cell wall, which is typically >80 nm thick. Therefore, if produced at an appropriate site (within the cell wall matrix or middle lamella), ^•^OH may have little effect on the protoplast. Moreover, in the case of a softening fruit pericarp, the cells involved are shortly destined to die in an animal’s gut, fulfilling their role in promoting seed dispersal. Therefore, any cellular damage caused to the ripe pericarp by ^•^OH is irrelevant; likewise in other short-lived tissues such as a lysing abscission zone or a rapidly expanding coleoptile.

### How could apoplastic ^•^OH be made *in vivo*?

The production of ^•^OH in plant cell walls most probably involves a Fenton-like reaction, whereby a transition metal ion in the reduced state reacts with hydrogen peroxide (H_2_O_2_):

$${\text{Cu}}^{+}+{\text{H}}_{2}{\text{O}}_{2}\to {\text{Cu}}^{\text{2}+}{+}^{•}\text{OH}+{\text{OH}}^{–}.$$

Two proposals have been considered: (1) the transition metal is the Fe of the haem group in peroxidase, which can be reduced by superoxide in a Haber–Weiss cycle (Chen and Schopfer, 1999; Liszkay *et al.*, 2003); and (2) a wall-bound transition metal (Cu and/or Fe) ion is reduced by apoplastic electron donors such as ascorbate (Fry, 1998; Vreeburg and Fry, 2005; Green and Fry, 2005; Kärkönen and Fry, 2006; Lindsay and Fry, 2007; Padu *et al.*, 2005). The H_2_O_2_ may be generated by wall-bound oxidases (Lane *et al.*, 1993; Asthir *et al.*, 2002; Kärkönen *et al.*, 2009) or superoxide dismutase (Yim *et al.*, 1990; Ogawa *et al.*, 1996; Kukavica *et al.*, 2009), or by non-enzymic reduction of O_2_ by ascorbate (Fry, 1998). Indeed, Dumville and Fry (2003) showed that the ability of cells in a tomato fruit to secrete ascorbate, and also the tissue’s Cu content, increased during ripening: effects that would be expected to favour *in-vivo* ^•^OH production.

### Is apoplastic ^•^OH made *in vivo*?

Cheng *et al.* (2008*a*) showed that, when homogenates of frozen banana pulp harvested at different stages of ripening were incubated for 12 h in phosphate buffer containing deoxyribose, *in-situ* generated ^•^OH (detected by its ability to oxidize the deoxyribose to dialdehyde products) increased in parallel with softening, suggesting that ripening may be associated with ^•^OH production in banana. Yang *et al.* (2008), who applied a similar method but with a shorter incubation period, also suggested that ^•^OH production increases prior to the initiation of banana fruit softening. However, in both these studies, the source of the ^•^OH in pulp was not clear and it could have been an artefact due to the homogenization, not reflecting reactions that occur *in vivo*.

^•^OH can cleave wall polysaccharides *in vitro*, but the question of whether ^•^OH is produced in the apoplast of living tissue and actually acts *in vivo* on wall polysaccharides in the manner proposed is still open, a major challenge being to detect such a short-lived free radical as ^•^OH in the walls of living cells. There are two possible experimental approaches: (1) infiltration into the apoplast of a membrane-impermeant ‘reporter’ compound that reacts with ^•^OH to give recognizable products (Kuchitsu *et al.*, 1995; Fry *et al.*, 2002; Schopfer *et al.*, 2002; Miller and Fry, 2004; Müller *et al.*, 2009; for a review, see Vreeburg and Fry, 2005); and (2) detection of the ‘collateral damage’ done to wall polysaccharides *in vivo* when attacked by apoplastic ^•^OH.

**Fig. 1.**
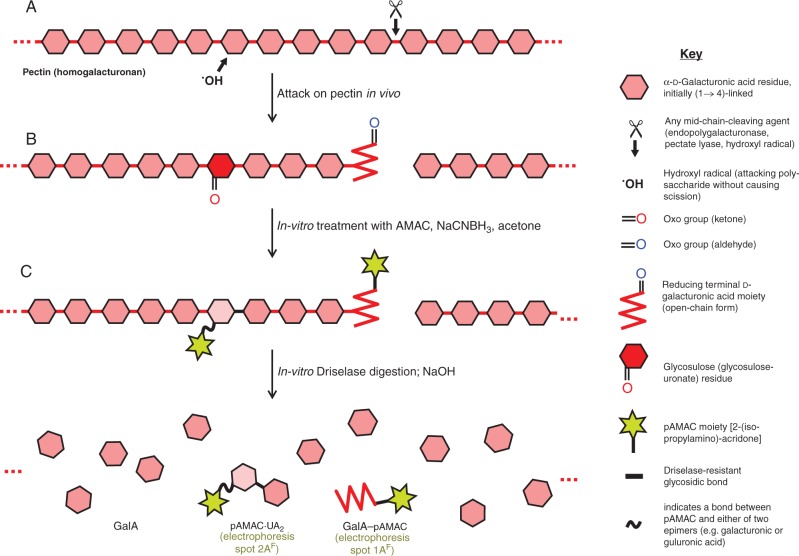
Schematic view of *in-vivo* attack on pectins and strategies used to detect it. (A) Part of a pectin (homogalacturonan) chain in the wall of a living fruit cell may be attacked either non-enzymically by a hydroxyl radical (^•^OH) or enzymically by endo-polygalacturonase or pectate lyase. Any of these three agents can cleave the backbone (e.g. at ✂), creating a new reducing terminus (shown in its non-cyclic form, and thus possessing an oxo group). In addition, ^•^OH can non-enzymically abstract an H atom (e.g. from C-2 or C-3 of a GalA residue) without causing chain scission; in an aerobic environment, this initial reaction leads to the formation of a relatively stable glycosulose residue possessing a mid-chain oxo group. (B) Wall material (AIR) is treated *in vitro* with AMAC, NaCNBH_3_ and acetone; oxo groups are reductively aminated to form yellow–green-fluorescing pAMAC conjugates. (C) The pAMAC-labelled homogalacturonan is then digested with Driselase, which hydrolyses all glycosidic bonds except any whose sugar residue carries a pAMAC group. The products tend to lactonize and are therefore briefly de-lactonized with NaOH before being fractionated. Further details of the reactions are given in figs 1 and 2 of Vreeburg *et al.* (2014).

The second approach is based on the fact that the ^•^OH radical cleaves polysaccharides by rather indiscriminate oxidative reactions. ^•^OH-driven polysaccharide scission, proposed to contribute to fruit softening, is accompanied by concurrent reactions that introduce relatively stable oxo groups into the polysaccharide (‘collateral damage’; Fig. 1A) without necessarily cleaving it (Zegota and von Sonntag, 1977; von Sonntag 1980; Vreeburg and Fry, 2005; Vreeburg *et al.*, 2014). Such oxo groups can serve as a chemical ‘fingerprint’ revealing recent ^•^OH attack in the cell walls of living cells. A polysaccharide usually has only a single oxo group (its reducing terminus), but ^•^OH attack generates oxo groups in mid-chain sugar residues, converting them to glycosulose residues (Vreeburg *et al.*, 2014). The proportion of such glycosulose residues (non-terminal oxo groups) per 1000 sugar residues would be a valuable measure of the extent of ^•^OH attack *in vivo*. Two methods are currently available for their detection:

#### Radiolabelling to detect glycosulose residues.

We have used reductive tritiation with NaB^3^H_4_ to detect oxo group formation in Fenton-treated soluble polysaccharides *in vitro* and in presumptively ^•^OH-exposed cell walls *in vivo* in ripening pears, germinating cress seeds and elongating maize coleoptiles (Fry *et al.*, 2001, 2002; Miller and Fry, 2001; Müller *et al.*, 2009; Iurlaro *et al.*, 2014). In this approach, each mid-chain glycosulose residue, formed by ^•^OH action, is reduced to yield a tritium-labelled simple sugar (aldose) residue – in some cases an aldose that does not frequently occur naturally. For example, ^•^OH attack at carbon-3 of a xylose residue in xyloglucan will introduce an oxo group which, when treated with NaB^3^H_4_ and then acid-hydrolysed, yields a mixture of [^3^H]xylose and [^3^H]ribose (the 3-epimer of xylose); the latter is not a natural constituent of xyloglucan and is therefore a highly diagnostic fingerprint (Miller and Fry, 2001).

#### Fluorescent labelling to detect glycosulose residues.

As an alternative to radiolabelling, we recently developed a method for fluorescently labelling mid-chain (^•^OH generated) oxo groups present in polysaccharides by reductive amination with 2-aminoacridone (AMAC) plus NaCNBH_3_, followed by *N*-isopropylation, to introduce fluorescent 2-isopropylaminoacridone (pAMAC) groups into the polysaccharide (Fig. 1B). The method was developed by model experiments on soluble pectic polysaccharides *in vitro* (Vreeburg *et al.*, 2014). Upon subsequent Driselase digestion (Fig. 1C), the mid-chain glycosulose residues, indicative of recent ^•^OH attack, were released as various products, of particular diagnostic value being pAMAC·disaccharide conjugates. [Note on nomenclature: The designation ‘sugar–pAMAC’ implies that the pAMAC group is linked to the former reducing group (carbon-1 in the case of an aldose) of the sugar, whereas ‘pAMAC·sugar’ indicates that the pAMAC group is attached to a different carbon of the sugar, whose C-1 remains unlabelled.] In contrast, the single reducing terminal oxo group of a poly- or oligosaccharide was released as a monosaccharide–pAMAC product. We now report the application of the pAMAC/Driselase method to demonstrate the changing abundance of ^•^OH-attacked polysaccharides in the cell walls of various contrasting fruits during ripening to give an indication of the involvement of hydroxyl radical attack in fruit softening.

## MATERIALS AND METHODS

### Materials

2-Aminoacridone was from Fluka (Dorset, UK). Driselase, from Sigma-Aldrich (Dorset, UK), was purified by ammonium sulphate precipitation and gel-permeation chromatography (Fry, 2000). The Luna C_18_ high-pressure liquid chromatography (HPLC) column [250 × 4·6 mm, 5 μm C_18_(2) 100 Å] was from Phenomenex (Cheshire, UK). The HPLC eluents were from VWR (Leicestershire, UK) or Fisher Scientific (Loughborough, UK). All other reagents were from Sigma-Aldrich or Fisher Chemicals. The PCE-PTR 200 penetrometer was from PCE Instruments UK Ltd (Southampton, UK).

Pear (*Pyrus communis* L.), mango (*Mangifera indica* L.), banana (hybrid based on *Musa acuminata* Colla), apple (*Malus pumila* Mill.), avocado (*Persea americana* Mill.), plum (*Prunus domestica* L.) and nectarine [*Prunus persica* (L.) Batsch] were from Sainsbury’s supermarket, Edinburgh; in each case, hard fruit not yet ready for eating were selected. Strawberry [*Fragaria* × *ananassa* (Weston) Duchesne ex Rosier (pro sp.)] was from Belhaven Fruit Farm, Dunbar, UK, and strawberry tree (*Arbutus unedo* L.) berries were generously provided by Sheffield Botanical Garden, UK. *Fragaria* and *Arbutus* fruit at three stages of ripening, distinguished by colour, were picked on the same day.

### Preparation of authentic sugar–pAMAC markers

Fluorescent markers for high-voltage paper electrophoresis (HVPE) and HPLC were prepared as before (Vreeburg *et al.*, 2014). In brief, a reducing sugar (0·4 μmol of dry glucose, GalA, GalA_2_, GalA_3_ or GalA_4_) was suspended in 40 μL of 0·1 m AMAC in dimethylsulphoxide (DMSO)/acetic acid/pyridine (17:2:1, by vol.) followed immediately by 40 μL of fresh aqueous 1 m NaCNBH_3_. After incubation of the mixture at 20 °C for 16 h, 2 μL of acetone and 40 μL of fresh 1 m NaCNBH_3_ were added and the mixture was incubated for another 1 h at 20 °C. The mixture was diluted with 5 vols of H_2_O and centrifuged (14 000 rpm, 10 min). The sugar–pAMAC product in the supernatant was purified on a C_18_ cartridge.

### Characterization of fruit softening and preparation of fruit AIR

Except for strawberry and *Arbutus*, freshly purchased hard fruits were stored in the dark in a wooden cupboard at room temperature in the laboratory. On selected days after purchase (when the fruits were hard, medium and soft, respectively), firmness was measured.

For firmness measurements, three individual fruit from each stage were randomly selected. Except with strawberry, the ‘skin’ was peeled. A 6 mm diameter penetrometer probe was positioned perpendicular to the peeled fruit surface, and the sensor was pressed down until it penetrated to the sensor’s indicator mark; the force shown on the display (in Newtons) was recorded.

A portion (10 g f. wt) of the edible part of each fruit was diced with a razor blade, immediately frozen with liquid N_2_ in a mortar, and ground to a fine powder with a pestle. Pre-cooled extractant (50 mL; ethanol/pyridine/acetic acid/water, 75:2:2:21 by vol., containing 10 mm Na_2_S_2_O_3_ to prevent Cu- or Fe-dependent ^•^OH production by Fenton reactions; Fry, 1998) was added and the mixture was ground again in the mortar for another 5 min. Finally, the whole homogenate was dispensed as 50 aliquots (each approx. 1·1–1·2 mL, equivalent to 200 mg f. wt of fruit tissue), which were stored at –80 °C.

*Arbutus* berries at different stages of ripening (orange, red and red–black) were immediately frozen at –80 °C and later homogenized as described above.

### pAMAC labelling of fruit AIR

All AMAC work was done under subdued red light; ice-cold solvents were used for the washing and precipitation steps. A portion of fruit AIR (alcohol-insoluble residue) suspension (≡200 mg fresh fruit tissue) was thawed and centrifuged. The pellet was washed twice with 75 % ethanol, blotted to remove free ethanol, and resuspended in 261 μL of a mixture comprising 45 μL of 0·5 % aqueous chlorobutanol, 5 μL of pyridine/acetic acid/water (2:2:1 by vol.; final pH approx. 4·0), 89 μL of DMSO containing 8·9 μmol AMAC and 61 μL of water containing 122 μmol freshly dissolved NaCNBH_3_; and the mixture was left for 20 h at 20 °C. Acetone (136 μmol) and an additional 122 μmol of fresh NaCNBH_3_ (61 μL of a 2 m aqueous solution) were added and the incubation was repeated for 16 h at 20 °C. To remove low molecular weight reagents and by-products, we added 1 mL of 96 % ethanol (to precipitate any water-soluble polysaccharides), pelleted the total polymers (at 12 000 *g* for 5 min), and washed the pellet twice with 1 mL of 75 % ethanol. The pellet was then re-suspended by shaking in 250 μL of pyridine/acetic acid/water (1:1:98 by vol.) for 10 min at 20 °C. The treatments with 96 and 75 % ethanol were repeated, and the final ethanolic pellet of pAMAC-labelled AIR was blotted to semi-dryness.

### Driselase digestion

The blotted pellet of pAMAC-labelled AIR was de-lactonized with 100 μL of 0·5 m NaOH (50 μmol) for 5 h at 20 °C, then buffered to pH 4·7 with two molar equivalents (5·75 μL) of acetic acid, washed with ice-cold 80 % ethanol, pelleted at 12 000 *g* for 5 min, blotted with filter paper and immediately treated with Driselase. [The de-lactonization step facilitated subsequent Driselase digestion.] The blotted, de-lactonized pellet of pAMAC-labelled AIR (equivalent to 200 mg of fresh weight fruit) was digested in 500 μL of 1 % partially purified Driselase in pyridine/acetic acid/0·5 % chlorobutanol, 1:1:98, by vol.) at 37 °C for 14 d, after which the solution was frozen at –20 °C.

### Purification of pAMAC-labelled products on a C_18_ column

A C_18_-silica cartridge (500 mg Supelco column; Sigma-Aldrich) was pre-conditioned with 2 vols of methanol then 2 vols of H_2_O. The soluble components of a whole Driselase digest (approx. 500 μL) were then loaded and the column was washed with 2 × 2 mL of H_2_O, after which bound solutes were eluted with 2 × 2 mL each of 10, 20, 30, 40 and 50 % (v/v) methanol. Each fraction was dried, redissolved in 50 μL of pyridine/acetic acid/water (1:1:98, by vol., pH approx. 4·7, containing 0·5 % chlorobutanol), and stored at –20 °C. Fractions exhibiting the characteristic yellow–green fluorescence of pAMAC groups were pooled for further analysis. Immediately before analysis by HVPE or HPLC, a portion was dried, de-lactonized in dilute NaOH (pH >11) at 20 °C for 10 min, and neutralized with acetic acid.

### High-voltage paper electrophoresis

Electrophoresis was conducted on Whatman No. 1 or No. 3 paper in a pH 6.5 buffer (pyridine/acetic acid/water, 33:1:300 by vol.) at 4·0 kV for 45–50 min. The papers were cooled with toluene. Methods and apparatus are described by Fry (2011*b*). After electrophoresis, the papers were dried and viewed under a 254 nm UV lamp and fluorescence was recorded photographically (Camlab DocIt system with LabWorks 4·6 software). Fluorescent spots on paper electrophoretograms were quantified with Image J (http://rsbweb.nih.gov/ij/) as described in the Supplementary Data Fig. S3.

### High-pressure liquid chromatography

The HPLC was conducted with a solvent flow rate of 1 mL min^–1^ at room temperature on a Luna C_18_ silica column with solvent A (500 mm acetic acid, adjusted to pH 5·0 with NaOH) and acetonitrile. All solvent compositions are given as percentage acetonitrile in solvent A, by vol. The column was pre-equilibrated for 30 min with 10 % acetonitrile. The injected sample (20 μL) was eluted with: 0–5 min, 10 % acetonitrile, isocratic; 5–15 min: 10–12·5 % acetonitrile, linear gradient; 15–30 min: 12·5 % acetonitrile, isocratic; 30–35 min, 12·5–15 % acetonitrile, linear gradient; 35–40 min, 15 % acetonitrile, isocratic; 40–50 min, 15–25% acetonitrile, linear gradient; 50–60 min, 25–10 % acetonitrile, linear gradient; 60–65 min, 10 % acetonitrile, isocratic. A fluorescence detector (RF 2000, Dionex) used excitation and emission wavelengths of 442 and 520 nm, respectively.

## RESULTS

### Application of pAMAC labelling to ripening fruit

**Fig. 2.**
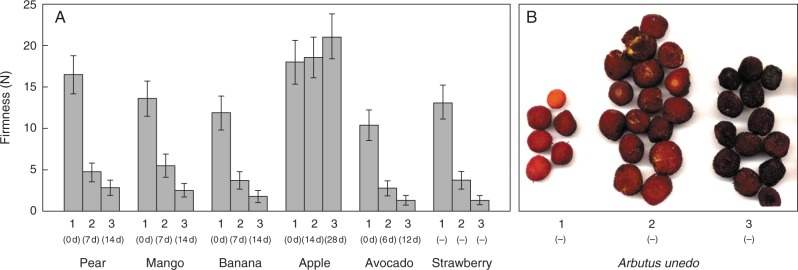
Softening of fruits at three stages of ripening. (A) Firmness data were obtained by penetrometer at three stages of ripening (1–3). Values are means (*n* = 3) ± s.e. Stages of softening were at various days after purchase as stated in parentheses. Strawberry and *Arbutus* fruit were chosen based on their colour, the different stages being picked on the same day. (B) No firmness readings for the *Arbutus* berries are available as they were frozen immediately after picking; their appearance is illustrated here.

Fruit firmness at three empirically defined stages of softening (hard, medium and soft) was measured – in most cases with a penetrometer (Fig. 2). The apples did not soften perceptibly within 1 month. All other species softened considerably between the three stages selected, although we did not quantify this for *Arbutus*.

Our approach for detecting ^•^OH attack on fruit cell walls at different stages of softening *in vivo* was based on the methodology developed for characterizing authentic ^•^OH-treated polysaccharides *in vitro* (Vreeburg *et al.*, 2014). AIR (cell wall-rich material) of unripe and ripe fruits was labelled with pAMAC, then exhaustively digested with Driselase. Fluorescent conjugates of negatively charged (mainly pectic) cell wall fragments, such as pAMAC·UA_2_ (dimer), would be strong evidence for ^•^OH-attacked pectins (Fig. 1). In contrast, GalA–pAMAC (monomer) would be derived from the pectins’ reducing terminus, and could be generated by any of three proposed agents (Fig. 1).

### Analysis of total Driselase digestion products of pAMAC-labelled fruit cell walls

#### Electrophoresis.

**Fig. 3.**
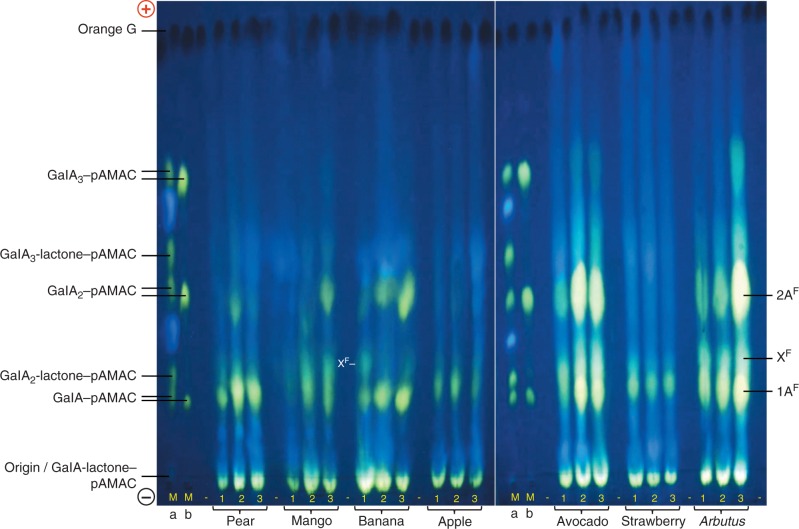
HVPE resolution of total Driselase digests of pAMAC-labelled AIR samples from seven fruit species. Fruit AIRs, each harvested at three stages of ripening (1–3; see Fig. 2), were successively treated with AMAC, acetone and Driselase (14 d); the pAMAC-labelled oligosaccharides generated were partially purified on a Supelco C_18_ cartridge column and de-lactonized in NaOH before electrophoresis. Each electrophoretogram loading was the products obtained from 20 mg f. wt of fruit tissue. Markers Ma and Mb are identical mixtures of acidic sugar–pAMAC conjugates before and after de-lactonization. Electrophoresis was at pH 6·5 and 4·0 kV for 45 min on Whatman No. 1 paper. Fluorescent spots were photographed under a 254-nm UV lamp. Orange G, loaded as a tracker between each fruit sample, shows up as a dark spot under UV. (+), anode; (–), cathode; –, blank loading.

Electrophoresis of the Driselase digestion products of pAMAC-labelled AIR (after de-lactonization) revealed at least two interesting, yellow–green-fluorescing, negatively charged spots (Fig. 3): **1A^F^**, co-migrating with the labelled monomer GalA–pAMAC; and **2A^F^**, approximately co-migrating with the labelled dimer GalA_2_–pAMAC. The anionic nature of **1A^F^** and **2A^F^** indicates that they were based on Driselase-digestible acidic sugar residues of the fruit cell walls, likely to be mainly GalA. Smaller amounts of putative acidic trimers were sometimes also observed, e.g. in avocado and *Arbutus*. An additional spot (**X^F^**), which fluoresced a less yellowish green, was seen in some species, especially in unripe banana.

In addition, strongly yellow–green-fluorescing neutral spots were present in all species; these spots could be based on any neutral, Driselase-releasable cell wall sugar residues. They showed no obvious changes in intensity throughout ripening.

Confirmatory replicate and additional studies, based on essentially the same technique as used for Fig. 3, are shown in Supplementary Data Figs S1 and S2.

Despite the approximate co-electrophoresis of **2A^F^** with GalA_2_–pAMAC, spot **2A^F^** cannot have contained GalA_2_–pAMAC itself since this compound is completely digested by Driselase under the conditions used. Instead, it is likely to have a constitution of the type pAMAC·UA-GalA (Fig. 1), a Driselase-resistant ‘fingerprint’ spot diagnostic of ^•^OH attack (Vreeburg *et al.*, 2014).

In pear, mango, banana, avocado and *Arbutus*, spot **1A^F^** appreciably increased in intensity (Fig. 3; Table 1), especially when stage 3 and stage 2 (soft and medium soft fruits) are compared with stage 1 (hard fruit). It also increased during softening in plum and nectarine (Table 1; Supplementary Data Fig. S1). On the other hand, the apple and strawberry AIR samples did not show any clear evidence of an increase in **1A^F^** at any stage.

**Table 1.** Relative abundance of the two major pAMAC-labelled anionic cell wall products at different stages of fruit softening

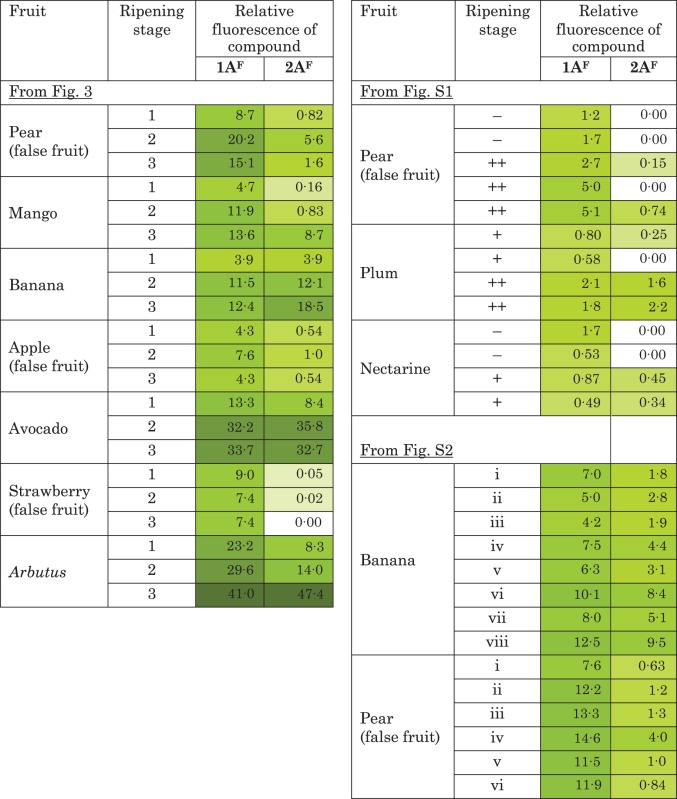										

Intensities were quantified on the electrophoretograms of de-lactonized samples as shown in Fig. 3 and Supplementary Data Figs S1 and S2.

Relative fluorescence is quoted in arbitrary units of area, as quantified in ImageJ by the method illustrated in Fig. S3.

The putative ‘fingerprint’ spot, **2A^F^**, was detected in all species, but was very weak in apple and strawberry. The yield of **2A^F^** increased during softening in mango, banana, avocado, *Arbutus*, plum and nectarine, and was always much fainter in hard, unripe fruit (Fig. 3; Table 1; Supplementary Data Figs S1 and S2). In pear, it was detected only at stage 2. The transient appearance of **2A^F^** in pear was confirmed in one repeat experiment (Fig. S2); it is possible that a brief period of high **2A^F^** yield was missed in an additional experiment (Fig. S1).

**Fig. 4.**
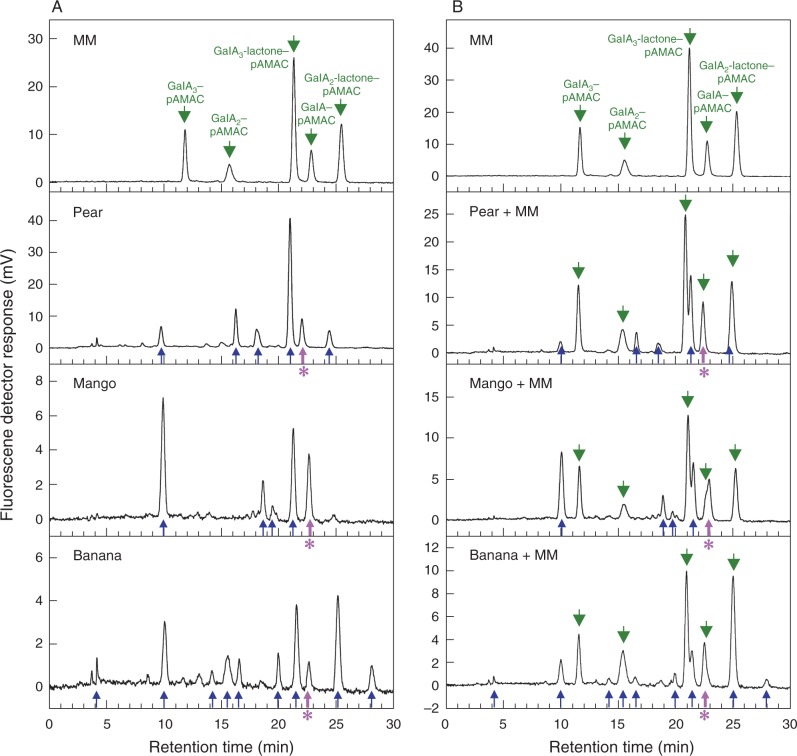
HPLC of total Driselase digests of pAMAC-labelled cell walls from three fruit species. AIRs from pear, mango and banana fruit (stages 3, 1 and 1, respectively) were treated with AMAC, acetone, Driselase, Supelco C_18_ and NaOH, all as in Fig. 3. Total fluorescent products (which will include conjugates of both neutral and acidic carbohydrates) were analysed by HPLC (A) before and (B) after addition of a marker mixture containing acidic sugar–pAMAC conjugates. Fluorescence detection was with excitation at 442 nm and emission at 520 nm. MM, marker mixture containing authentic acidic sugar–pAMAC conjugates. Green arrows, authentic sugar–pAMACs (including those added as a ‘spike’); blue arrows, unidentified peaks from fruit cell wall digests; thick purple arrows with asterisk, putative GalA–pAMAC from fruit cell wall digests.

Some samples, e.g. of pear and mango, revealed a weak spot that approximately co-migrated with GalA_3_-lactone–pAMAC (Fig. 3); however, HPLC showed that this specific compound was absent (Fig. 4; see below), as expected because it is Driselase digestible.

#### HPLC.

The presence of the reducing-end-labelled monomer, GalA–pAMAC, in the digests was supported by HPLC, which we performed on representative pear, mango and banana digests (stages 3, 1 and 1, respectively; without de-lactonization) both before and after spiking with a mixture of authentic sugar–pAMACs (Fig. 4). About 5–9 fluorescent peaks were detected by HPLC, with some differences between fruit species. In pear and banana, a compound was found (Fig. 4, thick purple arrow) which co-eluted with an internal marker of authentic GalA–pAMAC, supporting the idea that this was its identity. In mango, GalA–pAMAC partially overlapped with a compound of similar retention time (Fig. 4), which remains unidentified. Thus, in these fruit species, GalA–pAMAC was present, accounting for the **1A^F^** spot seen on electrophoretograms.

Concerning the labelled acidic dimers, HPLC of the pear and mango samples gave no peak exactly co-eluting with authentic internal marker GalA_2_–pAMAC. The pear sample gave a peak that eluted 1·2 min later than this marker. A small unidentified peak that did approximately co-elute with GalA_2_–pAMAC was found in the banana digest; however, this cannot have been GalA_2_–pAMAC itself because this substance is completely digested by Driselase under the conditions used (Vreeburg *et al.*, 2014).

### Further characterization of individual fluorescent spots by HPLC

**Fig. 5.**
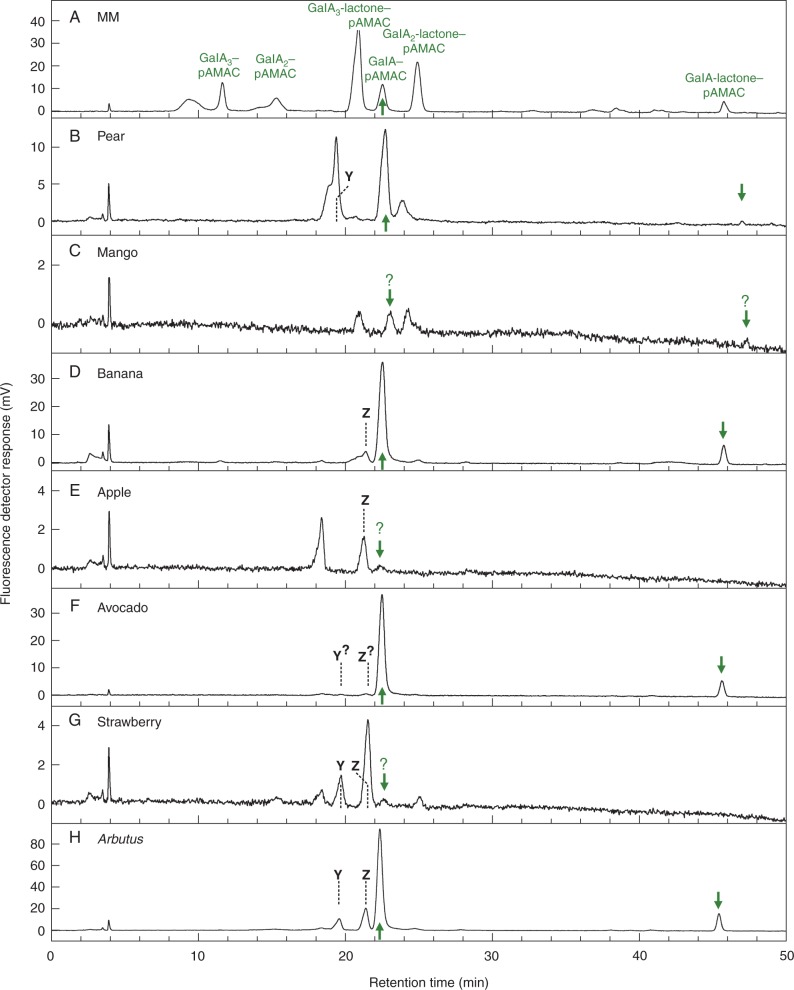
HPLC of the acidic monomer (**1A^F^**) spots from Driselase-digested pAMAC-labelled cell walls of seven fruit species. Each **1A^F^** spot (pooled for all three stages of development for each fruit; de-lactonized) shown in Fig. 3 was eluted from the electrophoretogram and analysed by HPLC. MM, marker mixture containing authentic acidic sugar–pAMAC conjugates. Arrows, putative GalA–pAMAC (and its lactone, which partially re-formed during elution from the electrophoretogram) from fruit cell wall digests. Dashed lines, compounds **Y** and **Z**, discussed in the text.

Samples of the material in the **1A^F^** zone were eluted from electrophoretograms and submitted to HPLC analysis, typically giving 2–4 peaks (Fig. 5). In at least four fruits (pear, banana, avocado and *Arbutus*), a major peak co-eluting with GalA–pAMAC was again detected, whereas in mango, apple and strawberry (the three species which showed the faintest **1A^F^** spots on the electrophoretogram), the corresponding peak was extremely minor. A small peak of GalA-lactone–pAMAC accompanied the GalA–pAMAC in pear, banana, avocado and *Arbutus*, supporting its identity since lactonization/de-lactonization is reversible and would be expected to occur between the electrophoresis step and the HPLC. Spot **1A^F^** from the electrophoretogram yielded in addition HPLC peaks other than GalA-lactone–pAMAC and GalA–pAMAC (Fig. 5), with some differences between fruit species. In particular, unidentified peaks **Y** and **Z** (see Fig. 5) were observed: **Y** in pear, **Z** in banana and apple, and both in *Arbutus*, strawberry and possibly avocado.

**Fig. 6.**
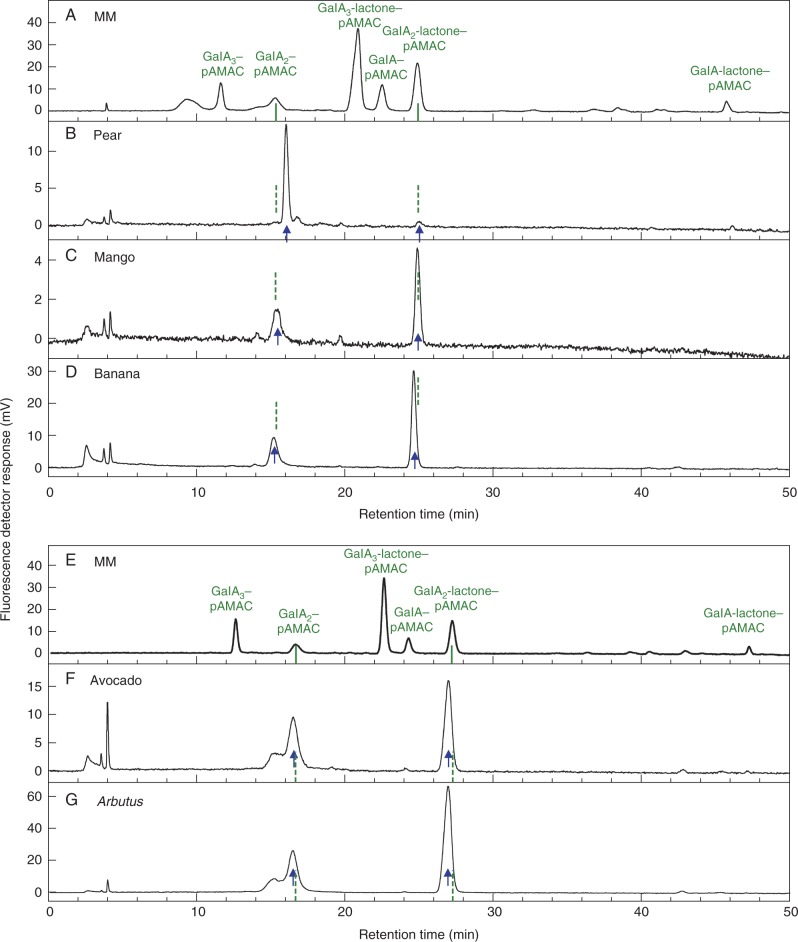
HPLC of the acidic dimer (**2A^F^**) spots from Driselase-digested pAMAC-labelled cell walls of five fruit species. **2A^F^** spots were eluted from a paper electrophoretogram (similar to that shown in Fig. 3 but derived from non-de-lactonized samples; all three ripening stages combined) and analysed by HPLC. MM, marker mixture containing authentic acidic sugar–pAMAC conjugates. Arrows, the proposed fingerprints for ^•^OH attack: pAMAC·UA-GalA and its lactone (rapidly re-formed during elution from the electrophoretogram) from fruit cell wall digests. Dashed lines, predicted position of authentic GalA_2_–pAMAC and GalA_2_-lactone–pAMAC, deduced from the marker run. The samples in the upper and lower graphs were run on different days, accounting for the slight discrepancy in marker retention times. Strawberry and apple were not included because they did not show any appreciable **2A^F^** spot in Fig. 3.

Spot **2A^F^**, deduced to be pAMAC·UA-GalA, which co-electrophoresed with authentic GalA_2_–pAMAC but was Driselase-stable, yielded HPLC peaks that only approximately co-eluted with authentic GalA_2_–pAMAC and GalA_2_-lactone–pAMAC in all species tested (Fig. 6). Both the acidic and lactone forms of the proposed pAMAC·UA-GalA were present in these eluates because of their interconversion, which is more rapid than in the case of the GalA_2_–pAMAC ↔ GalA_2_-lactone–pAMAC interconversion (Vreeburg *et al.*, 2014).

**Fig. 7.**
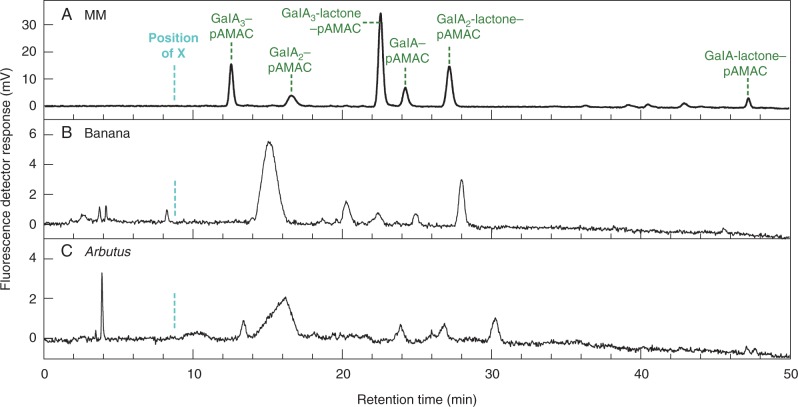
HPLC of the acidic unknown (**X^F^**) spots from Driselase-digested pAMAC-labelled cell walls of banana and *Arbutus*. The **X^F^** spot (similar to that shown in Fig. 3 but from a non-de-lactonized sample) for stage-1 banana and *Arbutus* was eluted from an electrophoretogram and analysed by HPLC. MM, marker mixture containing authentic acidic sugar–pAMAC conjugates. The cyan dashed line indicates the approximate retention time of unknown ‘**X**’ (relative to the GalA_3_–pAMAC peak) seen in the products obtained from *in-vitro* ^•^OH-treated pectin (Vreeburg *et al.*, 2014). Green dashed lines indicate the authentic markers.

Electrophoretogram spot **X^F^**, a greenish-fluorescing compound migrating slightly faster than authentic GalA_2_-lactone–pAMAC and observed in banana (and possibly *Arbutus*), was resolved by HPLC into several small peaks (Fig. 7). These peaks, however, did not match the HPLC peak ‘**X**’ found after pAMAC labelling of *in-vitro* ^•^OH-treated pectin (Vreeburg *et al.*, 2014), even though both have similar migration and fluorescence properties on the electrophoretogram. Both **X** and **X^F^** remain to be identified.

## DISCUSSION

A fluorescent fingerprinting method, recently developed for demonstrating hydroxyl radical attack on polysaccharides *in vitro* (Vreeburg *et al.*, 2014), has now been applied to the cell wall polysaccharides of several fruit species at different stages of softening, providing useful information on ^•^OH attack *in vivo*. The fluorescent labelling procedure can yield information comparable with a radiolabelling approach used earlier (Fry *et al.*, 2001, 2002; Müller *et al.*, 2009; Iurlaro *et al.*, 2014), and the two approaches are largely interchangeable. However, the fluorescent pAMAC group introduced into the polysaccharides in the new method provides a means of further characterization of these cell wall components by means of a wide variety of accessible chromatography and electrophoresis techniques, including fluorophore-assisted electrophoresis (Goubet *et al.*, 2002). Specifically, pAMAC introduces a pH-dependent charge into the ^•^OH-attacked, plant-derived polymer residue, which facilitates further characterization of the products. Radiolabelling is generally more sensitive and is very straightforward to quantify, but not all laboratories are authorized to use it.

At least two informative fluorescent spots (**1A^F^** and **2A^F^**) were visualized on electrophoretograms (Fig. 3). Spot **1A^F^**, including predominantly GalA–pAMAC (Fig. 1), increased in intensity between stages 1 and 3 of softening in most fruits (Table 1). This would correspond to an increasing number of d-GalA reducing termini during fruit ripening, which could be caused by the pectic polysaccharide-cleaving actions of not only ^•^OH (as a result of attack at C-1 or C-4 of homogalacturonan – see fig. 1 of Vreeburg *et al.*, 2014) but also endo-PG and/or pectate lyase. Both endo-PG and pectate lyase, proposed fruit softening agents, can attack a homogalacturonan chain, creating one new reducing terminus per cleavage event and this reducing terminus would become pAMAC labelled. Several studies have reported increases in endo-PG activity (though seldom definitively distinguished from pectate lyase activity) in pear (Pressey and Avants, 1976), banana (Pathak and Sanwall, 1998; Ali *et al.*, 2004) and avocado (Huber and O’Donoghue, 1993). Increasing pectate lyase activity has been measured during ripening in banana (Payasi and Sanwal, 2003; Payasi *et al.*, 2006). In addition, pectate lyase mRNA accumulation was reported in several ripening fruits including banana (Dominquez-Puigjaner *et al.*, 1997; Marín-Rodríguez *et al.*, 2003) and mango (Chourasia *et al.*, 2006). Therefore, spot **1A^F^** obtained from fruit AIR was not exclusive evidence of ^•^OH attack, but may offer a valuable fingerprint indicating the total pectic chain scission occurring *in vivo*.

Spot **2A^F^** was concluded to be a Driselase limit digestion product of the type pAMAC·UA-GalA (Fig. 1), i.e. a ‘fingerprint’ indicating recent *in-vivo* mid-chain ^•^OH attack. The precise chemical identity of the compound(s) present in spot **2A^F^** has not been established. **2A^F^** clearly did not include the reducing-terminus-labelled disaccharide, GalA_2_–pAMAC, since this compound does not withstand 14 d of Driselase treatment (Vreeburg *et al.*, 2014), and GalA_2_–pAMAC was not observed in pear and mango by HPLC analysis (Fig. 4). It probably includes pAMAC·GalA-GalA and/or its 2-, 3- and 4-epimers (pAMAC·taluronate-GalA, pAMAC·guluronate-GalA and pAMAC·glucuronate-GalA, respectively). We would expect all these structures to be Driselase resistant because the range of activities present in Driselase probably does not include α-taluronidase, α-guluronidase and α-glucuronidase, and because the pAMAC group would block the action of α-galacturonidase.

The intensity of spot **2A^F^** increased appreciably as hard fruit (stage 1) matured into softer fruit (stages 2 and 3) in mango, banana, avocado and *Arbutus*. The observation in banana may possibly be related to the ripening-dependent increase in the reported ability of banana fruit homogenates to generate ‘endogenous’ ^•^OH post-mortem (Cheng *et al.*, 2008*a*; Yang *et al.*, 2008). In pear, the increase in spot **2A^F^** was transient, peaking in stage 2; this suggests that the glycosulose residues from which **2A^F^** is generated (Fig. 1) were unstable *in vivo*. A related observation in pear (increase in ^3^H-labelled products released when fruit cell walls were NaB^3^H_4_ labelled and then Driselase digested) was reported by Fry *et al.* (2001), where the unidentified ^3^H-labelled products were proposed to be ‘fingerprints’ of ^•^OH attack.

The increase in yield of **2A^F^** during softening depended on the type of fruit under consideration. In true fruits (those whose edible portion is derived from the ovary wall; including mango, banana, avocado, *Arbutus*, plum and nectarine), there was an increase in **2A^F^** that correlated with softening. In contrast, it showed little if any increase in apple or strawberry and increased only transiently in pear, which are all false fruits. In false fruits, the edible tissue is derived from the receptacle, not the ovary wall. Therefore, differences in developmental origin of the edible tissue may dictate the mechanism adopted for cell wall modification during ‘fruit’ softening.

### Conclusions

It was reported nearly 40 years ago that in pear fruit, endogenous peroxides (and thus potentially also ^•^OH generated from them) correlate with softening (Brennan and Frenkel, 1977). Later it was found that the polysaccharides of softening pears exhibit radiochemical ‘fingerprints’ diagnostic of recent ^•^OH attack (Fry *et al.*, 2001). Furthermore, of two investigated cultivars of muskmelon, the one whose microsomal membranes produced less ^•^OH *in vitro* had a longer shelf-life (Lacan and Baccou, 1998). Taken together, the available evidence supports the view that fruit softening, often viewed as broadly a ‘hydrolytic’ phenomenon, is at least partly ‘oxidative’ – a suggestion raised by Brennan and Frenkel (1977) but often ignored. We hope that interest in this concept will be revived by the present study and explored in greater depth. Although several of the fluorescent ‘fingerprint’ compounds were not fully identified in the present study and deserve further analysis, our new fluorescent labelling method will provide useful information and can be used in conjunction with other approaches to add to our knowledge and understanding of the occurrence and rate of ^•^OH attack relative to endo-PG and pectate lyase action in fruit cell walls.

## SUPPLEMENTARY DATA

Supplementary data are available online at www.aob.oxfordjournals.org and consist of the following. Figure S1: electrophoretic resolution of total Driselase digests of pAMAC-labelled cell walls from three fruit species. Figure S2: electrophoretic resolution of total Driselase digests of pAMAC-labelled cell walls from banana and pear. Figure S3: method for quantification of fluorescent spots on paper electrophoretograms.

Supplementary Data
