# Supplementary material for: Pectic polysaccharides are attacked by hydroxyl radicals in ripening fruit: evidence from a fluorescent fingerprinting method
Source: Ann Bot. 2016 Feb 9;117(3):441–55. doi: 10.1093/aob/mcv192 (PMC4765547; doi:10.1093/aob/mcv192)
Supplement: Supplementary Data [file supp_117_3_441__index.html]

Pectic polysaccharides are attacked by hydroxyl radicals in ripening fruit: evidence from a fluorescent fingerprinting method — Pectic polysaccharides are attacked by hydroxyl radicals in ripening fruit: evidence from a fluorescent fingerprinting method — Supplementary Data 

# Pectic polysaccharides are attacked by hydroxyl radicals in ripening fruit: evidence from a fluorescent fingerprinting method

## Supplementary Data

files

- Supplementary Data - pptx file
