# Supplementary material for: Pectic polysaccharides are attacked by hydroxyl radicals in ripening fruit: evidence from a fluorescent fingerprinting method
Source: Ann Bot. 2016 Feb 9;117(3):441–55. doi: 10.1093/aob/mcv192 (PMC4765547; doi:10.1093/aob/mcv192)
Supplement: Supplementary Data [file supp_mcv192_aob-15643-s01.pptx]

## Slide 1
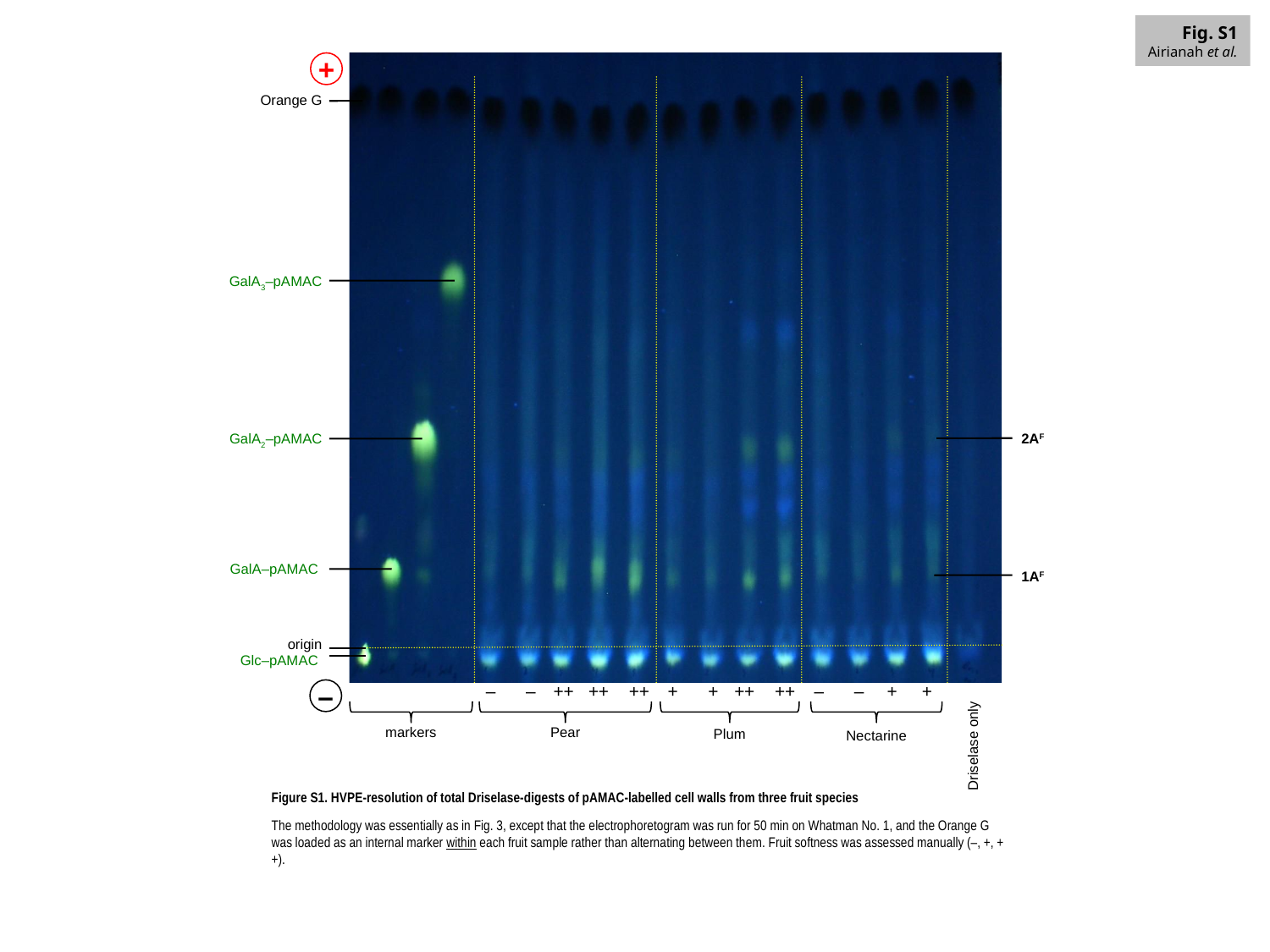

Fig. S1
Airianah et al.
+
Orange G
GalA3–pAMAC
2AF
GalA2–pAMAC
GalA–pAMAC
1AF
origin
Glc–pAMAC
–
–
–
++
++
++
+
+
++
++
–
–
+
+
markers
Pear
Plum
Nectarine
Driselase only
Figure S1. HVPE-resolution of total Driselase-digests of pAMAC-labelled cell walls from three fruit species
The methodology was essentially as in Fig. 3, except that the electrophoretogram was run for 50 min on Whatman No. 1, and the Orange G was loaded as an internal marker within each fruit sample rather than alternating between them. Fruit softness was assessed manually (–, +, ++).

## Slide 2
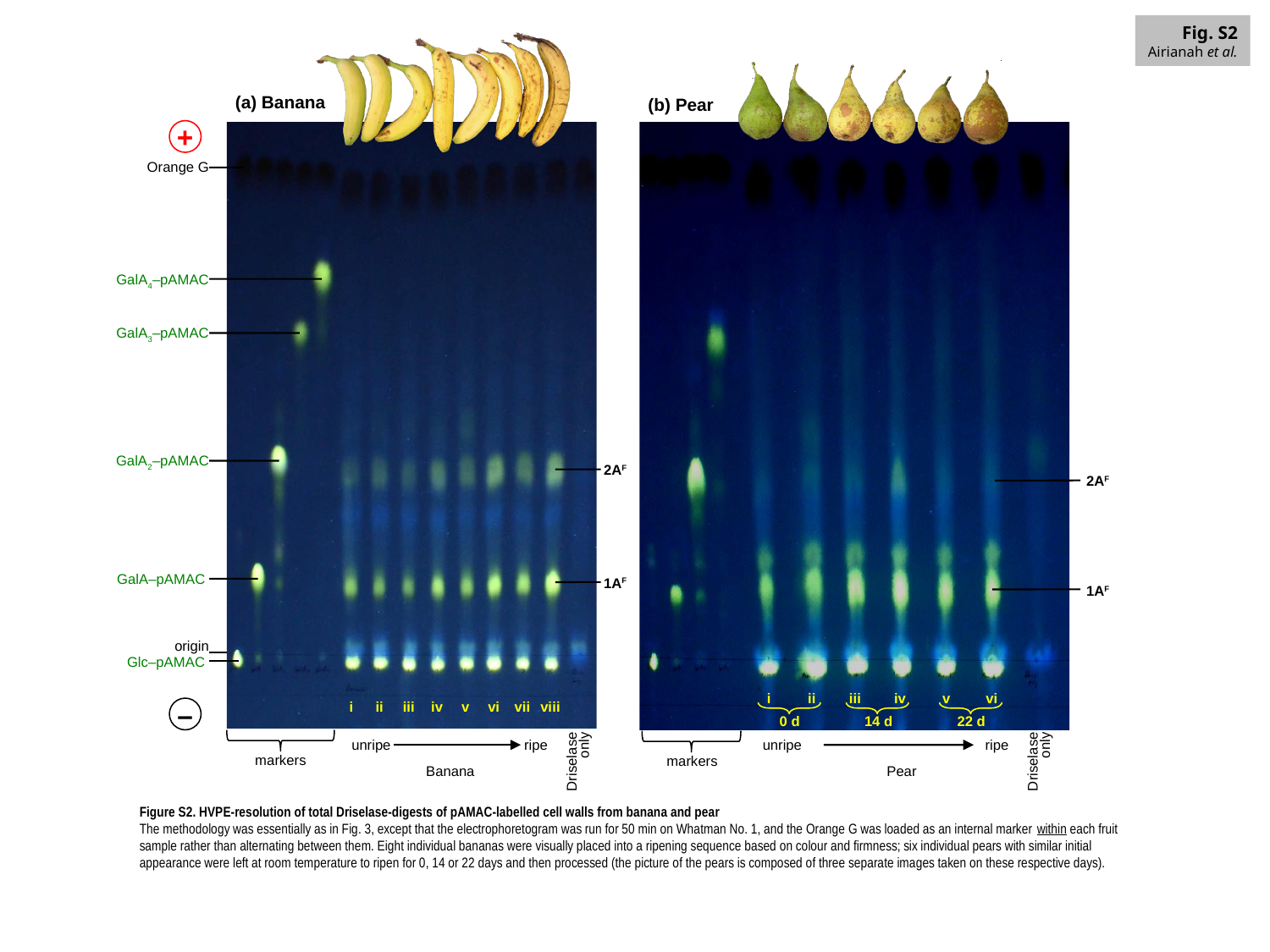

Fig. S2
Airianah et al.
(a) Banana
(b) Pear
+
Orange G
GalA4–pAMAC
GalA3–pAMAC
GalA2–pAMAC
2AF
2AF
1AF
GalA–pAMAC
1AF
origin
Glc–pAMAC
i
ii
iii
iv
v
vi
–
i
ii
iii
iv
v
vi
vii
viii
0 d
14 d
22 d
unripe
ripe
unripe
ripe
Driselase
only
Driselase
only
markers
markers
Banana
Pear
Figure S2. HVPE-resolution of total Driselase-digests of pAMAC-labelled cell walls from banana and pear
The methodology was essentially as in Fig. 3, except that the electrophoretogram was run for 50 min on Whatman No. 1, and the Orange G was loaded as an internal marker within each fruit sample rather than alternating between them. Eight individual bananas were visually placed into a ripening sequence based on colour and firmness; six individual pears with similar initial appearance were left at room temperature to ripen for 0, 14 or 22 days and then processed (the picture of the pears is composed of three separate images taken on these respective days).

## Slide 3
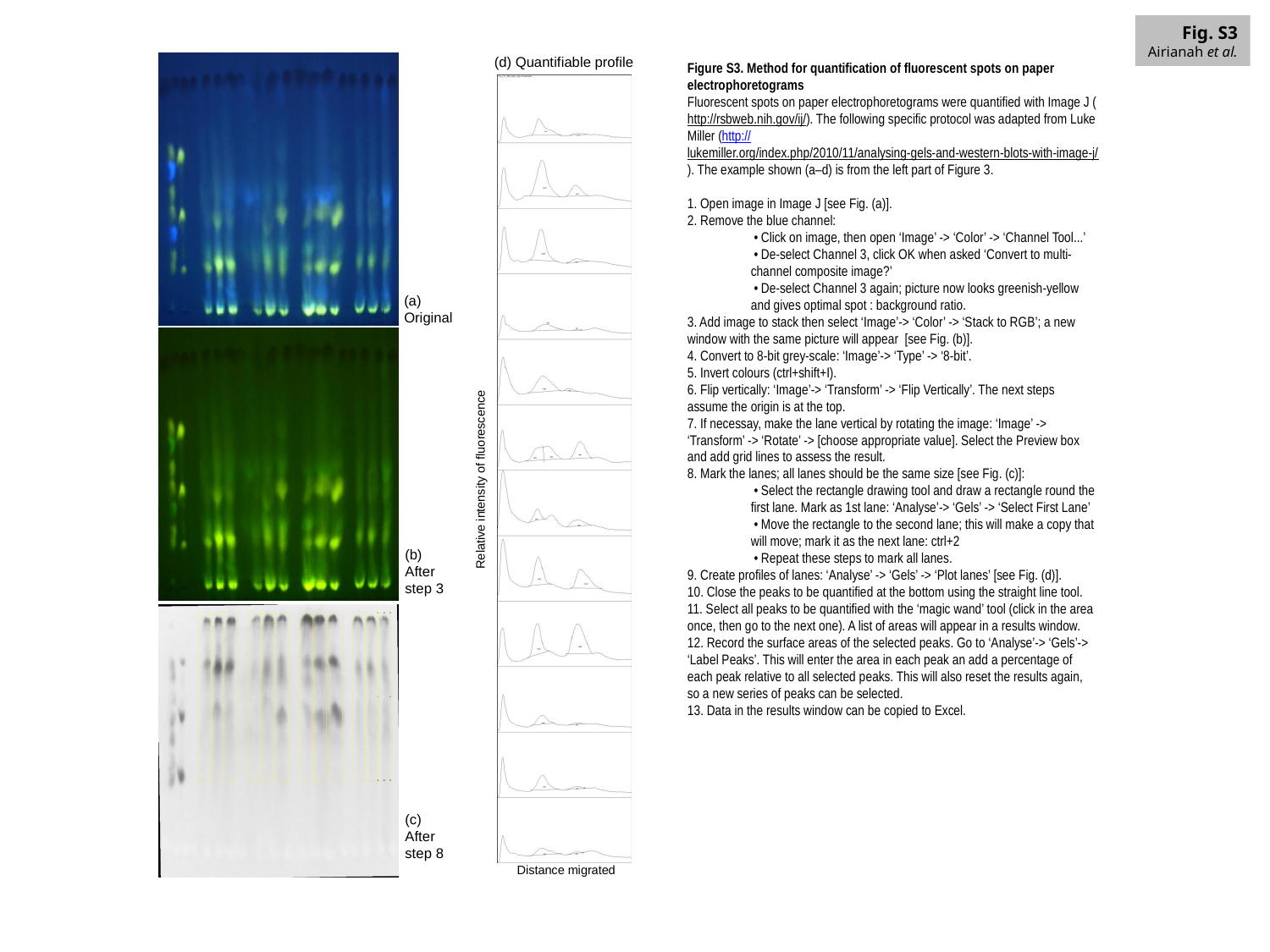

Fig. S3
Airianah et al.
(a)
Original
(b)
After
step 3
(c)
After
step 8
(d) Quantifiable profile
Relative intensity of fluorescence
Distance migrated
Figure S3. Method for quantification of fluorescent spots on paper electrophoretograms
Fluorescent spots on paper electrophoretograms were quantified with Image J (http://rsbweb.nih.gov/ij/). The following specific protocol was adapted from Luke Miller (http://lukemiller.org/index.php/2010/11/analysing-gels-and-western-blots-with-image-j/). The example shown (a–d) is from the left part of Figure 3.
1. Open image in Image J [see Fig. (a)].
2. Remove the blue channel:
 • Click on image, then open ‘Image’ -> ‘Color’ -> ‘Channel Tool...’
 • De-select Channel 3, click OK when asked ‘Convert to multi-channel composite image?’
 • De-select Channel 3 again; picture now looks greenish-yellow and gives optimal spot : background ratio.
3. Add image to stack then select ‘Image’-> ‘Color’ -> ‘Stack to RGB’; a new window with the same picture will appear [see Fig. (b)].
4. Convert to 8-bit grey-scale: ‘Image’-> ‘Type’ -> ‘8-bit’.
5. Invert colours (ctrl+shift+I).
6. Flip vertically: ‘Image’-> ‘Transform’ -> ‘Flip Vertically’. The next steps assume the origin is at the top.
7. If necessay, make the lane vertical by rotating the image: ‘Image’ -> ‘Transform’ -> ‘Rotate’ -> [choose appropriate value]. Select the Preview box and add grid lines to assess the result.
8. Mark the lanes; all lanes should be the same size [see Fig. (c)]:
 • Select the rectangle drawing tool and draw a rectangle round the first lane. Mark as 1st lane: ‘Analyse’-> ‘Gels’ -> ‘Select First Lane’
 • Move the rectangle to the second lane; this will make a copy that will move; mark it as the next lane: ctrl+2
 • Repeat these steps to mark all lanes.
9. Create profiles of lanes: ‘Analyse’ -> ‘Gels’ -> ‘Plot lanes’ [see Fig. (d)].
10. Close the peaks to be quantified at the bottom using the straight line tool.
11. Select all peaks to be quantified with the ‘magic wand’ tool (click in the area once, then go to the next one). A list of areas will appear in a results window.
12. Record the surface areas of the selected peaks. Go to ‘Analyse’-> ‘Gels’-> ‘Label Peaks’. This will enter the area in each peak an add a percentage of each peak relative to all selected peaks. This will also reset the results again, so a new series of peaks can be selected.
13. Data in the results window can be copied to Excel.
